# 1444. Influenza Surveillance in the Veterans Health Administration (VHA): 2020-2021 Season

**DOI:** 10.1093/ofid/ofab466.1636

**Published:** 2021-12-04

**Authors:** Cynthia A Lucero-Obusan, Patricia Schirmer, Gina Oda, Mark Holodniy

**Affiliations:** Department of Veterans Affairs, Palo Alto, California

## Abstract

**Background:**

Veterans Health Administration’s (VHA) large elderly population is at higher risk for influenza complications, including hospitalization and death. Herein we summarize VHA’s national annual surveillance data for seasonal influenza activity and vaccinations.

**Methods:**

Influenza telephone triage, influenza-like-illness (ILI) encounters and antiviral prescriptions plus outpatient visits, laboratory testing, hospitalizations and deaths for influenza were obtained from VHA data sources (9/27/20-5/22/21) and compared to prior years and CDC FluView data. Influenza vaccinations were captured from 8/1/2020. Vaccination rates were calculated based on VHA users during the fiscal year.

**Results:**

Surveillance metrics are presented (Table). Vaccinations were decreased and ILI was below average (0.3%-0.7% per week). Activity was highest 2020 Weeks 46-47 but remained low the entire season with no distinct peak seen, matching national influenza activity (Fig. 1). Testing revealed 161 influenza positives from 440,553 tests performed (0.04%). Hospitalizations among laboratory-confirmed cases were similar to the prior season (16% vs 17%). Median length of stay (6 days) and deaths (17, 12%) were increased over prior seasons. Among 15 deaths where results were available, 4 had Influenza A, 10 had Influenza B and 1 had Influenza A+B. Nine were co-infected with COVID-19. Total influenza positives, outpatient visits, hospitalizations and antiviral use were extremely low compared to all prior season where national VHA data has been analyzed (Table, Fig. 2).

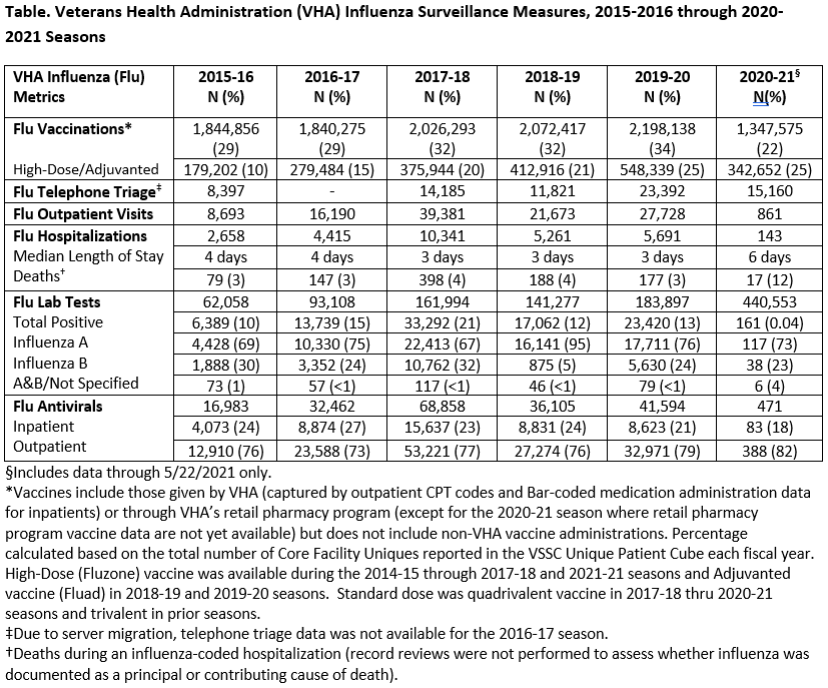

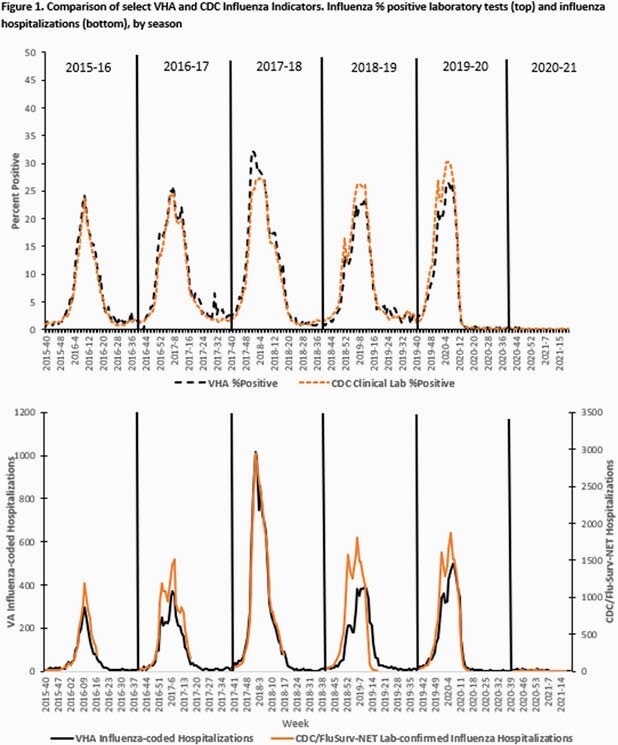

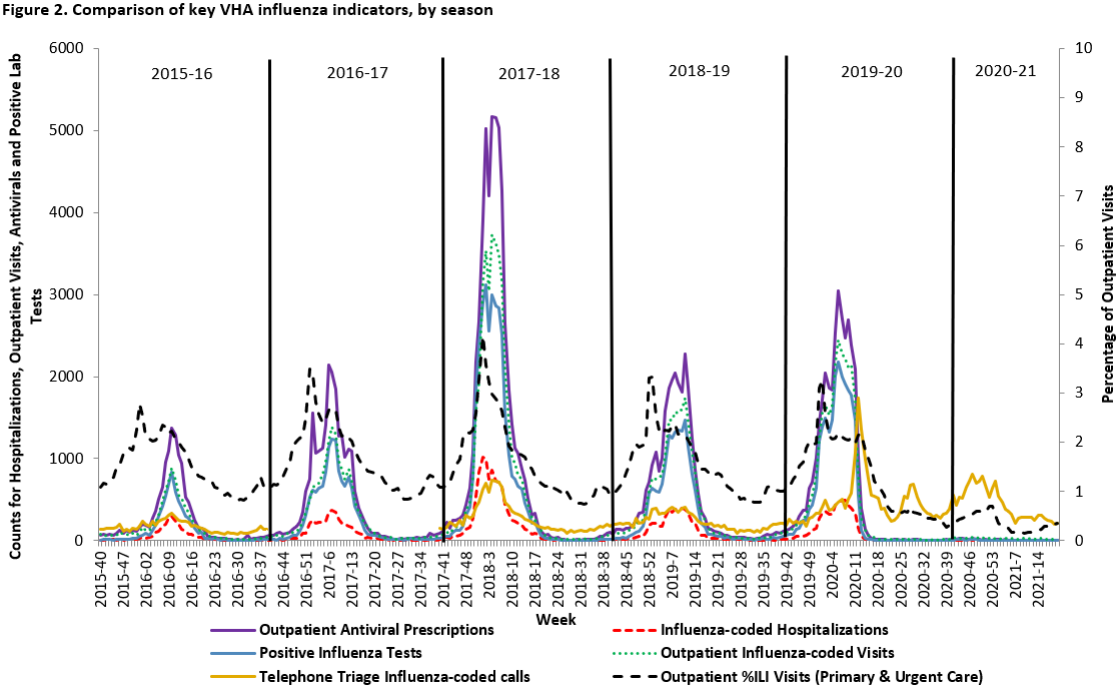

**Conclusion:**

Overall, influenza vaccination levels were decreased although percent receiving high-dose formulation was stable. Despite lower vaccination rates, the 2020-21 influenza season was of historically low activity, even with markedly increased testing performed in the setting of multiplex tests for influenza with COVID-19. Deaths were primarily seen with Influenza B and among those co-infected with COVID-19. This may also have contributed to increased length of stay. VHA influenza activity continues to track closely with national CDC data and may have been impacted by mitigation measures used to contain COVID-19, which were likely effective in curbing influenza activity.

**Disclosures:**

**All Authors**: No reported disclosures

